# Control of respiratory drive by extracorporeal CO_2_ removal in acute exacerbation of COPD breathing on non-invasive NAVA

**DOI:** 10.1186/s13054-019-2404-y

**Published:** 2019-04-23

**Authors:** Christian Karagiannidis, Stephan Strassmann, Sarah Schwarz, Michaela Merten, Eddy Fan, Jennifer Beck, Christer Sinderby, Wolfram Windisch

**Affiliations:** 1 Department of Pneumology and Critical Care Medicine, Cologne-Merheim Hospital, ARDS and ECMO Centre, Kliniken der Stadt Köln gGmbH, Witten/Herdecke University Hospital, Ostmerheimer Strasse 200, D-51109 Cologne, Germany; 2 Interdepartmental Division of Critical Care Medicine, University of Toronto, Toronto, Canada; 3 Keenan Research Centre for Biomedical Science and Department of Critical Care Medicine, St. Michael’s Hospital, Toronto, Canada; 4 Department of Pediatrics, University of Toronto, Toronto, Canada; 5 Department of Medicine, University of Toronto, Toronto, Canada; 6 Extracorporeal Life Support Program, Toronto General Hospital, Toronto, Canada

**Keywords:** Non-invasive ventilation, Extracorporeal carbon dioxide removal, COPD, Exacerbation, Neurally adjusted ventilatory assist

## Abstract

**Background:**

Veno-venous extracorporeal CO_2_ removal (vv-ECCO_2_R) and non-invasive neurally adjusted ventilator assist (NIV-NAVA) are two promising techniques which may prevent complications related to prolonged invasive mechanical ventilation in patients with acute exacerbation of COPD.

**Methods:**

A physiological study of the electrical activity of the diaphragm (Edi) response was conducted with varying degrees of extracorporeal CO_2_ removal to control the respiratory drive in patients with severe acute exacerbation of COPD breathing on NIV-NAVA.

**Results:**

Twenty COPD patients (SAPS II 37 ± 5.6, age 57 ± 9 years) treated with vv-ECCO_2_R and supported by NIV-NAVA were studied during stepwise weaning of vv-ECCO_2_R. Based on dyspnea, tolerance, and blood gases, weaning from vv-ECCO_2_R was successful in 12 and failed in eight patients. Respiratory drive (measured via the Edi) increased to 19 ± 10 μV vs. 56 ± 20 μV in the successful and unsuccessful weaning groups, respectively, resulting in all patients keeping their CO_2_ and pH values stable. Edi was the best predictor for vv-ECCO_2_R weaning failure (ROC analysis AUC 0.95), whereas respiratory rate, rapid shallow breathing index, and tidal volume had lower predictive values. Eventually, 19 patients were discharged home, while one patient died. Mortality at 90 days and 180 days was 15 and 25%, respectively.

**Conclusions:**

This study demonstrates for the first time the usefulness of the Edi signal to monitor and guide patients with severe acute exacerbation of COPD on vv-ECCO_2_R and NIV-NAVA. The Edi during vv-ECCO_2_R weaning was found to be the best predictor of tolerance to removing vv-ECCO_2_R.

**Electronic supplementary material:**

The online version of this article (10.1186/s13054-019-2404-y) contains supplementary material, which is available to authorized users.

## Introduction

Neurally adjusted ventilatory assist (NAVA) is a mode of mechanical ventilation which applies assist in proportion to the electrical activity of the diaphragm (Edi). NAVA and non-invasive NAVA (NIV-NAVA) have been shown to improve patient-ventilator interaction, both in terms of timing and responding to increased demand, regardless of leaks [1–4]. NAVA can be considered to act as an external respiratory muscle, sharing the work in synchrony with the patient’s own inspiratory muscles, in order to maintain adequate minute ventilation. An important feature of NAVA is that despite its ability to reduce respiratory drive with increasing assist, full respiratory muscle unloading does not eliminate the Edi completely. In fact, about 30–50% of the initial Edi remains during the highest levels of assist [4–7].

Veno-venous extracorporeal CO_2_ removal (vv-ECCO_2_R) is part of a spectrum of techniques [8] that can rapidly remove CO_2_ and reverse respiratory acidosis, thereby reducing the central respiratory drive at lower than normal minute ventilation [9–16]. In patients with COPD, vv-ECCO_2_R in combination with NIV has the potential to prevent the complications associated with the use of invasive mechanical ventilation. One major drawback of invasive mechanical ventilation in COPD compared to non-invasive ventilation is the higher rate of nosocomial infections (i.e., ventilator associated pneumonia, central venous line, and urinary tract infections [17]) and prolonged weaning with high morbidity and mortality [18, 19]. Two recent case-controlled studies by Braune et al. [20] and Del Sorbo et al. [21] demonstrated the feasibility of avoiding invasive mechanical ventilation with vv-ECCO_2_R, although substantial device-related side effects and the subsequent need for intubation due to worsening oxygenation [20] were observed. However, we lack rigorous data on the efficacy of ECCO_2_R in patients with acute exacerbation of COPD beyond these proof-of-concept studies [11–14, 22–25].

The combination of NAVA and vv-ECCO_2_R offers a novel approach to treating severely hypercapnic patients, as it allows for simultaneous monitoring of respiratory drive via the Edi and offers both the support of the respiratory muscles with reversal of hypercapnia through the extracorporeal system. In fact, the combination of invasive NAVA and ECMO with even higher CO_2_ removal capacity is synergistic when used together in patients with severe ARDS [26]. During support with a constant NAVA level, reductions in sweep gas flow from a level that maintained normal PaCO_2_, increased both Edi and peak inspiratory airway pressure. Furthermore, Pisani et al. [27] and Diehl et al. [28] demonstrated a reduction of the work of breathing by ECCO_2_R in COPD patients in the weaning process from invasive mechanical ventilation.

The application of vv-ECCO_2_R in combination with awake NIV-NAVA has not been explored in patients with severe COPD exacerbation and combined hypercapnic/hypoxemic respiratory failure. Knowing that there is a direct relationship between Edi and CO_2_ load in healthy subjects and outpatients [29, 30], we hypothesized that Edi would respond to ECCO_2_R and could be an important predictor of weaning from extracorporeal systems in awake patients. The aim of the present study, therefore, was to evaluate the association between Edi and tolerance to weaning from vv-ECCO_2_R in patients with severe acute exacerbation of COPD supported with NIV-NAVA.

## Methods

The Ethics Committee of the University of Witten/Herdecke provided approval for this study. Written informed consent was obtained from all enrolled patients. Patients were recruited over a period of more than 2 1/2 years. The study was registered retrospectively (German Clinical Trials Register/DRKS 00012737).

### Patient inclusion criteria

As a part of our routine clinical practice, intubated COPD patients failing a spontaneous breathing trial (SBT) and remaining acidotic (pH 7.25–7.35) despite invasive mechanical ventilation or if they required re-intubation within 48 h after extubation were placed on vv-ECCO_2_R prior to study inclusion. Patients were approached for inclusion after being extubated to NIV-NAVA and were clinically stable (i.e., pH ≥ 7.40, minimal secretions, low dose or no catecholamines, adequate level of consciousness). Further details on patient characteristics and vv-ECCO_2_R are given in the online data supplement.

### vv-ECCO_2_R

Standard configuration of the system consisted of a 19Fr/38 cm femoral-draining cannula and a 17 Fr/15 cm inlet-flow cannula (Maquet, Rastatt, Germany). All vv-ECCO_2_R runs were performed with the Cardiohelp system (HLS set advanced 5.0). The use of a vv-ECCO_2_R system with a surface area of 1.3 m^2^ allowed for an increase in blood flow of up to 2–3 L/min if necessary, to remove more CO_2_ and lower the respiratory drive, or even improve oxygenation. Standard anticoagulation with heparin was used to target an aPTT of 1.8–2.0-fold of the normal range.

### Main intervention (weaning from vv-ECCO_2_R) and measurements

**Fig. 1:**
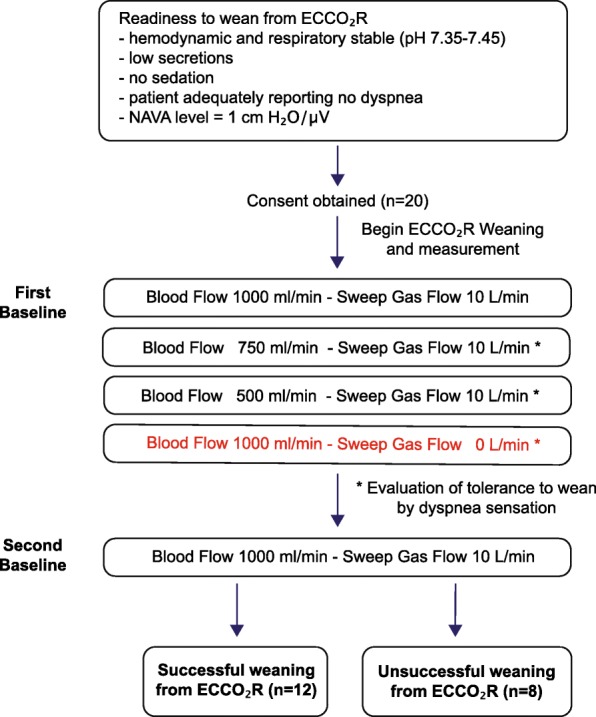
Flow chart of inclusion criteria, study protocol, and outcome. See the main text for details

Readiness to wean from vv-ECCO_2_R was assessed clinically according to the criteria noted in Fig. 1. On the day weaning readiness was reached, the intervention phase of the study was carried out by the following protocol: NIV-NAVA level was set to 1.0 cm H_2_O/μV, the peak pressure limit to 27–30 cmH_2_O, and positive end-expiratory pressure (PEEP) to 6–7 cmH_2_O. FiO_2_ was titrated to maintain a PaO_2_ of 60–80 mmHg. Once achieved, FiO_2_ was kept stable throughout the weaning process, even if PaO_2_ increased. Subsequently, the following five 30-min conditions were studied: (i) an initial “first” baseline period, with vv-ECCO_2_R settings of 1000 mL/min blood flow and 10 L/min sweep gas flow (O_2_); (ii) reduced vv-ECCO_2_R blood flow to 750 mL/min; (iii) reduced vv-ECCO_2_R blood flow to 500 mL/min; (iv) a pause of sweep gas flow; and (v) return to a second baseline period. If the patients did not tolerate reduced blood flow, a pause of sweep gas flow, or were clearly stating to stop the weaning protocol, the settings were returned to baseline.

After the second baseline, patients were decannulated if they reported no dyspnea while having the sweep gas off and if pH values were ≥ 7.35. Dyspnea was classified as either “acceptable dyspnea” (which lead to removal of the system) or “very severe dyspnea,” (no removal of the system). Arterial blood gases were obtained at the end of each condition. Extracorporeal CO_2_ elimination was measured in the exhaust of the oxygenator as previously described [31].

### Data analysis

Data were collected and analyzed off-line as previously described [26]. Mean values for peak Edi, peak airway inspiratory pressure (PIP), and expiratory tidal volume (Vt), breathing frequency, and minute ventilation were calculated for the whole period of each condition. Neuro-ventilatory efficiency (NVE) was calculated by dividing Vt by Edi as described before [32]. Rapid shallow breathing index (RSBI) was calculated by dividing breathing frequency by tidal volume.

Continuous variables are reported as mean (± standard deviation). One-way ANOVA followed by Dunnett’s multiple comparisons test, unpaired *t* test, and receiver-operating characteristic (ROC) analysis was performed as appropriate. Further details on data analysis are given in the online data supplement.

## Results

Twenty patients were studied with a maximum follow-up period of 2 years (Table 1). Prior to hospitalization, 40% of the patients had been on home NIV, and 60% had been on long-term oxygen treatment. All patients had severe emphysema on computed tomography (CT) scans with concomitant bronchiolitis and/or infiltration. A diagnosis of severe COPD was confirmed by history, CT scan, and flow pattern on mechanical ventilation by two experienced pulmonologists from our lung center. Before study inclusion, all patients received invasive mechanical ventilation following initial NIV failure, with mean PaCO_2_ 96.2 ± 20.5 mmHg and mean pH 7.19 ± 0.06. Clinically, vv-ECCO_2_R was initiated with a mean blood flow of 2.1 ± 0.8 L/min and a mean sweep gas flow of 4.5 ± 1.9 L/min. The relatively higher blood flow rate applied was mainly necessary due to worsening of oxygenation in the initial phase. Blood flow was gradually reduced over time, depending on the patient’s gas exchange and clinical course with daily evaluation for the possibility of weaning from the extracorporeal system.

**Table 1:** Patients baseline and mortality

	*N* = 20
Basic characteristics	
Age [years]	57 ± 9
Female/male	13/7
SAPS II at admission	37 ± 6
Body mass index	27.1 ± 7.6
Home NIV before	8/20
Long-term oxygen therapy before	12/20
Known history of severe COPD	20/20
ECCO_2_R	
pH before ECCO_2_R (invasive mechanical ventilation)	7.19 ± 0.06
PaCO_2_ before ECCO_2_R [mmHg]	96.2 ± 20.5
HCO3^−^ before ECCO_2_R [mmol/l]	30.5 ± 6.2
ECCO_2_R blood flow day 1 [l/min]	2.1 ± 0.8
Sweep gas flow day 1 [l/min]	4.5 ± 1.9
ECCO_2_R blood flow day before protocol [l/min]	1.0 ± 0.2
Sweep gas flow day before protocol [l/min]	10 ± 0.5
Major bleeding events	
Retroperitoneal hematoma	2
Hematothorax	1
Pulmonary bleeding	2
Mortality	
90-day mortality	15%
180-day mortality	25%

Prior to study inclusion, vv-ECCO_2_R was associated with major bleeding events in five patients (two patients with spontaneous retroperitoneal hematoma, one patient with hemothorax without prior intervention, two patients with pulmonary bleeding). These events prolonged intensive care unit (ICU) length of stay but did not affect mortality. Mortality was 15% at 90 days and 25% at 180 days (Table 1 and Additional file 1: Figure S4). Additional file 1: Figure S4 shows the Kaplan-Meier curves for survival (*N* = 20) after 1 and 2 years post-study.

Two experienced intensivists judged whether patients who were awake and cooperative were ready to wean, according to clinical criteria (detailed description in the online data supplement). During the study, 12 patients could be successfully weaned from the extracorporeal system during the first attempt, while eight patients remained on the system due to severe patient-reported dyspnea while undergoing the weaning protocol. No patient had to be re-intubated. From eight patients failing the primary weaning attempt, seven could be successfully weaned from vv-ECCO_2_R on their daily assessment at a later date, while one patient died in hospital on vv-ECCO_2_R (Fig. 1).

During the first baseline (blood flow of 1000 mL/min with a sweep gas flow of 10 L/min), mean extracorporeal CO_2_ removal was 129 ± 21 mL/min in patients successfully weaned and 142 ± 46 mL/min in patients with unsuccessful weaning (NS) (Additional file 2: Figure S1 A-B).

**Fig. 2:**
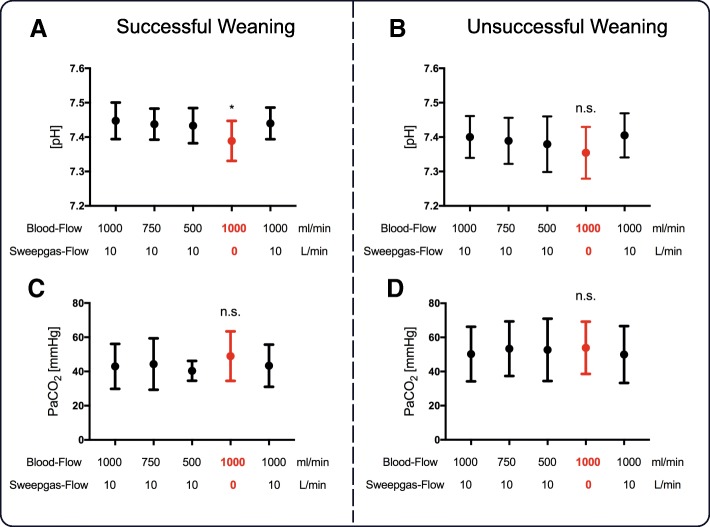
pH value (**a**, **b**) and PaCO_2_ (**c**, **d**) in groups of successful and unsuccessful vv-ECCO_2_R weaning readiness test. From left to right, values obtained during first baseline (blood flow = 1000 mL/min and sweep gas flow = 10 L/min) and at blood flow to 750 mL/min and 500 mL/min with maintained sweep gas flow, followed by turning off sweep gas flow with 1000 mL/min blood flow and a second repeated baseline. For detailed description, see the main text. *Difference compared to baseline (*P < 0.05, **P < 0. 01, ***P < 0.001)

**Fig. 3:**
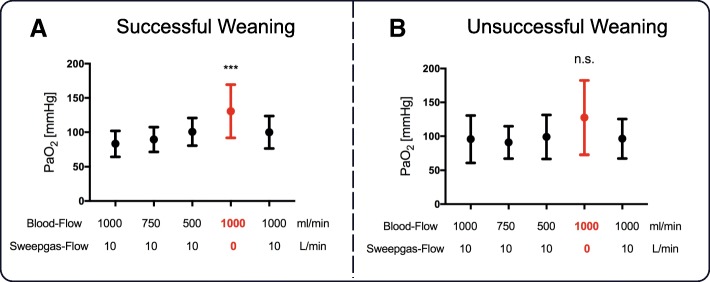
PaO_2_ (**a**, **b**) in groups of successful and unsuccessful vv-ECCO_2_R weaning readiness test. From left to right, values obtained during first baseline (blood flow = 1000 mL/min and sweep gas flow = 10 L/min) and at blood flow to 750 mL/min and 500 mL/min with maintained sweep gas flow, followed by turning off sweep gas flow with 1000 mL/min blood flow and a second repeated baseline. For detailed description, see the main text. *Difference compared to baseline (*P < 0.05, **P < 0. 01, ***P < 0.001)

While breathing on a NIV-NAVA level of 1 cmH_2_O/μV, most patients maintained their PaCO_2_ and pH values within normal range regardless of changes in blood flow. However, turning off sweep gas flow resulted in a slight decrease in pH (Fig. 2a–d). Of note, oxygenation increased significantly in patients with successful weaning when sweep gas flow was paused (Fig. 3a, b).

**Fig. 4:**
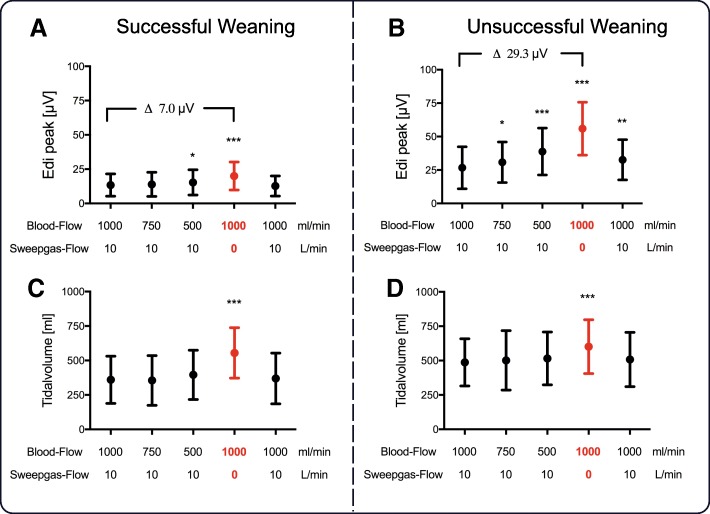
Peak diaphragm electrical activity (Edi) (**a** and **b**) and tidal volume (**c** and **d**) in groups of successful and unsuccessful vv-ECCO2R weaning readiness test. From left to right, values obtained during first baseline (blood flow = 1000 mL/min and sweep gas flow = 10 L/min) and at blood flow to 750 mL/min and 500 mL/min with maintained sweep gas flow, followed by turning off sweep gas flow with 1000 mL/min blood flow and a second repeated baseline. For detailed description, see the main text. *Difference compared to baseline (*P < 0.05, **P < 0.01, ***P < 0.001)

The Edi at first baseline was significantly lower in patients with successful as compared to unsuccessful weaning from vv-ECCO_2_R (13.4 ± 8.1 μV vs. 26.7 ± 15.7 μV, *P* < 0.001) (Fig. 4a, b). In both groups, reducing blood flow increased Edi; however, this increase was more pronounced in the unsuccessful group. Compared to first baseline, turning off the sweep gas flow increased Edi by a significantly smaller amount in the successful as compared to unsuccessful weaning group (7.0 μV vs. 29.3 μV, *P* < 0.001). The Edi without sweep gas flow was significantly lower in the successful group (19 ± 10 μV vs. 56 ± 20 μV, *P* < 0.001) (Fig. 4a, b).

Tidal volume (Vt) at first baseline was significantly lower in the successful group (360 ± 171 mL [5.7 mL/kg predicted body weight (PBW)] vs. 487 ± 172 mL [8.1 mL/kg PBW], *P* < 0.001) and was not affected by changes in blood flow (Fig. 4c, d). Tidal volume increased by a significantly lower amount in the successful group after sweep gas flow was turned off (555 ± 183 mL [8.8 mL/kg PBW] vs. 601 ± 196 mL [9.8 ml/kg PBW], *P* < 0.05).

**Fig. 5:**
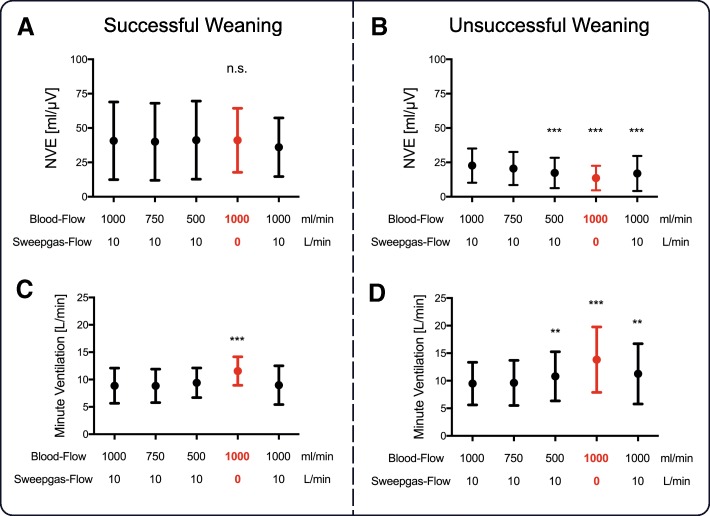
**a**–**d** Neuro-ventilatory efficiency (NVE) and minute ventilation in groups of successful and unsuccessful vv-ECCO_2_R weaning readiness test. From left to right, values obtained during first baseline (blood flow = 1000 mL/min and sweep gas flow = 10 L/min) and at blood flow to 750 mL/min and 500 mL/min with maintained sweep gas flow, followed by turning off sweep gas flow with 1000 mL/min blood flow and a second repeated baseline. For detailed description, see the main text. *Difference compared to baseline (*P < 0.05, **P < 0.01, ***P < 0.001)

The mean neuro-ventilatory efficiency (NVE) at first baseline was significantly higher in patients with successful vv-ECCO_2_R weaning (40.7 ± 28.2 vs. 22.7 ± 12.5 ml/μV, *P* < 0.001) (Fig. 5a, b). In the unsuccessful group, NVE was significantly reduced at 500 mL blood flow and further decreased to 13.6 ± 8.9 ml/μV when sweep gas flow was turned off. Further data on PIP, breathing frequency, and minute ventilation are given in the online data supplement and Fig. 5c, d/Additional file 3: Figure S3.

The area under the receiver-operating characteristic curve (AUROC) analysis was 0.95 (95% CI 0.93–0.97) for Edi, 0.89 (95% CI 0.86–0.92) for NVE, 0.77 (95% CI 0.72–0.81) for PIP, 0.57 (95% CI 0.51–0.63) for minute ventilation and Vt, 0.54 (95% CI 0.49–0.59) for rapid shallow breathing index (RSBI) under NIV-NAVA, and 0.53 (95% CI 0.48–0.59) for breathing frequency (Additional file 4: Figure S2).

## Discussion

Our study supports the use of vv-ECCO_2_R in combination with NIV-NAVA to control respiratory drive in patients with acute exacerbations of COPD. Our results show that the Edi is not only a gauge to monitor the effect of CO_2_ removal but also a predictor of tolerance to weaning from vv-ECCO_2_R. Patients who fail to wean from vv-ECCO_2_R have significantly greater respiratory drive (Edi) and dyspnea than patients who can be successfully weaned from extracorporeal support. Taken together, our study demonstrates proof of concept of implementing vv-ECCO_2_R in combination with NIV-NAVA for the treatment of severe acute exacerbation of COPD.

### Predictors of weaning from vv-ECCO_2_R

Our study included non-sedated patients with severe COPD initially reporting no dyspnea when supported on NIV-NAVA and vv-ECCO_2_R and whose success or failure of system weaning was based on perceived dyspnea and pH values. Although dyspnea could be considered a subjective measurement, it could discriminate between patients who tolerated removal of sweep gas flow and was very closely predicted by Edi (AUROC 0.95). The reason behind the high degree of discrimination was that halting the sweep gas flow resulted in extremely high Edi values in the unsuccessful group, almost four times that of the successful group. Also, in previous studies, Edi and NVE during spontaneous breathing tests have been described as good predictors for extubation readiness [32–35]. We did not measure intrinsic PEEP at baseline, and patients that eventually failed weaning from ECCO_2_R may have been the ones with higher intrinsic PEEP at baseline. However, our results of the high predictive value of the Edi are consistent with a recent study demonstrating that CO_2_ washout by nasal cannula oxygen therapy also decreases post-extubation neuro-ventilatory drive and work of breathing in patients with COPD [36].

### Ability to maintain PaCO_2_ and pH

The Edi represents the central respiratory drive [29, 37, 38] and is affected by a multitude of neural receptors [39]. The Edi increases in response to CO_2_ loading [29] and to increased respiratory muscle loading [40]. The Edi also increases with respiratory muscle weakness [41] induced by hyperinflation, typically observed in COPD patients [38, 42, 43]. NAVA supports the inspiratory muscles in response to the Edi and complements the patient’s effort to adjust ventilation. As NAVA is a proportional assist mode, its relative sharing of force generation with the patient remains constant throughout inspiration, regardless if they have a lower or higher Edi. Since NAVA is a proportional mode and all the patients received the same NAVA level (1 cmH_2_O), the degree of support was variable, depending on patient’s Edi. Thus, NAVA enhances the patient’s “force-generating” capacity to unload the respiratory muscles, which can help to reduce the patient’s sensation of respiratory effort. However, in some patients, the central respiratory drive is affected by metabolic or irritative reasons, leading sometimes to uncontrolled breathing independent of the level of support.

Similar to the present study, a previous small study in severe ARDS patients on NAVA showed that termination of sweep gas flow led to an increased Edi and required adjustments to ventilation to maintain PaCO_2_ and pH [22]. Of note, the stepwise reduction in blood flow rate had only a minor impact on CO_2_ removal capacity due to the large membrane lung surface area of our ECCO_2_R system. However, the present study shows clearly that the combination vv-ECCO_2_R and NIV-NAVA makes it possible to detect if the patient’s respiratory drive is “uncontrollable” with just mechanical ventilation. The essential need to preserve PaCO_2_ and pH was supported by the fact that 8 out of 20 patients who failed removal of sweep gas flow responded with an extreme respiratory drive (Edi > 50 μV). When respiratory drive was uncontrollable, it was possible to remove the CO_2_ load and “need-to-breathe-more” sensation using extracorporeal CO_2_ elimination.

### Respiratory parameter response

Our results showed that the successful group had lower baseline values for Edi, PIP, V_t_, and that these parameters were only affected by removal of sweep gas flow, which despite a low increase in Edi, increased PIP, V_t_, and minute ventilation to values similar to those of the unsuccessful group. The tolerance of dyspnea in the successful group shows that they had enough reserve to allow removal of the extracorporeal system. Moreover, the maintenance of NVE suggests the lack of an impact on respiratory muscle function and/or mechanics. In the unsuccessful group, both reducing blood flow and removal of sweep gas flow led to a significant increase in respiratory drive as evidenced by the increased Edi. As the Edi was extremely high in the unsuccessful group, their PIP reached the maximum pressure limit, and we can only speculate that the results could have been different if the upper pressure limit has been increased. We hypothesize that despite high Edi and low NVE, the unsuccessful group would not benefit from more non-invasive assist to unload the diaphragm and thus would never have been able to remain on NIV-NAVA unless also supported by the extracorporeal system. Of note, Edi in the unsuccessful group remained increased during the second baseline, which could be attributed to increased CO_2_ production by the respiratory muscles [28].

### Clinical considerations

The CO_2_ removal capacity during treatment was comparable to the CO_2_ removal capacity observed in animal experiments using newer oxygenators [31, 44] reflecting the effectiveness of the system used in the present study, even after several days of treatment in patients.

In the present study, five major bleeding events occurred during vv-ECCO_2_R, which prolonged ICU length of stay but had no impact on mortality. An interesting observation was the increase in PaO_2_ during vv-ECCO_2_R weaning. Global vasodilation of the pulmonary vessels following CO_2_ removal improving right heart function [45] has been suggested to increase the shunt perfusion [45]. Moreover, ECCO_2_R affects mainly PCO_2_ in the VCO_2_/VO_2_ ratio, which could lead to worsening of the oxygenation [46].

## Conclusions

Our study demonstrates the feasibility of implementing vv-ECCO_2_R in combination with NIV-NAVA for the treatment of severe acute exacerbation of COPD. Patients who fail to wean from vv-ECCO_2_R have significantly greater respiratory drive (Edi) and dyspnea than patients who can be successfully weaned from extracorporeal support. A high Edi signal of > 50 μV when pausing the extracorporeal system with a stable pH value of 7.4 could be predictive for unsuccessful weaning of vv-ECCO_2_R. Randomized controlled trials of vv-ECCO_2_R and NIV-NAVA vs. conventional invasive mechanical ventilation in patients with severe acute exacerbations of COPD are needed to confirm these findings and the efficacy of this approach.

## Additional files

Additional file 1: **Figure S4.** Kaplan-Meier curve of all 20 patients treated with vv-ECCO_2_R and NIV-NAVA in severe exacerbation of COPD within 2 years. A 90- and 180-day mortality remained low with 15% and 25%, respectively. (PDF 946 kb)

Additional file 2: **Figure S1.** Veno-venous extracorporeal CO_2_ elimination. First baseline extracorporeal CO_2_ removal was 129 ± 21 ml/min in the successful group (panel A) and 142 ± 46 mL/min in the unsuccessful group. From left to right, values obtained during first baseline (blood flow = 1000 mL/min and sweep gas flow = 10 L/min) and at blood flow to 750 mL/min and 500 mL/min with maintained sweep gas flow, followed by turning off sweep gas flow with 1000 mL/min blood flow and a second repeated baseline. For detailed description, see the main text. *Difference compared to baseline (*P < 0.05, **P < 0.01, ***P < 0.001). (PDF 1887 kb)

Additional file 3: **Figure S3.** Peak inspiratory airway pressure (PIP) and breathing frequency in (Fb) groups of successful and unsuccessful vv-ECCO_2_R weaning readiness test. From left to right, values obtained during first baseline (blood flow = 1000 ml/min and sweep gas flow = 10 L/min) and at blood flow to 750 ml/min and 500 mL/min with maintained sweep gas flow, followed by turning off sweep gas flow with 1000 mL/min blood flow and a second repeated baseline. For detailed description, see the main text. *Difference compared to baseline (*P < 0.05, **P < 0.01, ***P < 0.001). (PDF 2824 kb)

Additional file 4: **Figure S2.** Receiver-operating characteristic curve (ROC) analysis for the groups of successful and unsuccessful vv-ECCO_2_R weaning readiness test (*N* = 20) that were ventilated on NIV-NAVA when sweep gas flow was turned off. ROC analysis was applied for peak diaphragm electrical activity (Edi), neuro-ventilatory efficiency (NVE), peak airway pressure (PIP), rapid shallow breathing index (RSBI), minute ventilation, breathing frequency, and tidal volume. For detailed description see the main text. (PDF 2343 kb)

## Abbreviations

ECMO: Extracorporeal membrane oxygenation
Edi: Electrical activity of the diaphragm
NAVA: Neurally adjusted ventilatory assist
NIV-NAVA: Non-invasive NAVA
vv-ECCO_2_R: Veno-venous extracorporeal CO_2_ removal
