# Supplementary figures and images for: Control of respiratory drive by extracorporeal CO2 removal in acute exacerbation of COPD breathing on non-invasive NAVA
Source: Crit Care. 2019 Apr 23;23:135. doi: 10.1186/s13054-019-2404-y (PMC6480839; doi:10.1186/s13054-019-2404-y)

Supplemental Figure 4

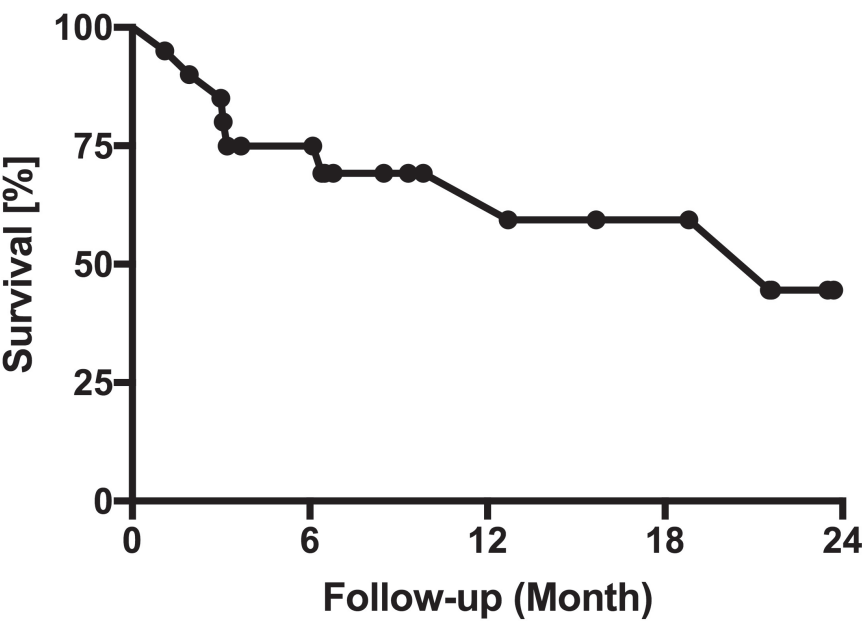

Supplement: Supplementary file 1 — Figure S4. Kaplan-Meier curve of all 20 patients treated with vv-ECCO2R and NIV-NAVA in severe exacerbation of COPD within 2 years. A 90- and 180-day mortality remained low with 15% and 25%, respectively. (PDF 946 kb) [file 13054_2019_2404_MOESM1_ESM.pdf]

## Supplemental Figure 1

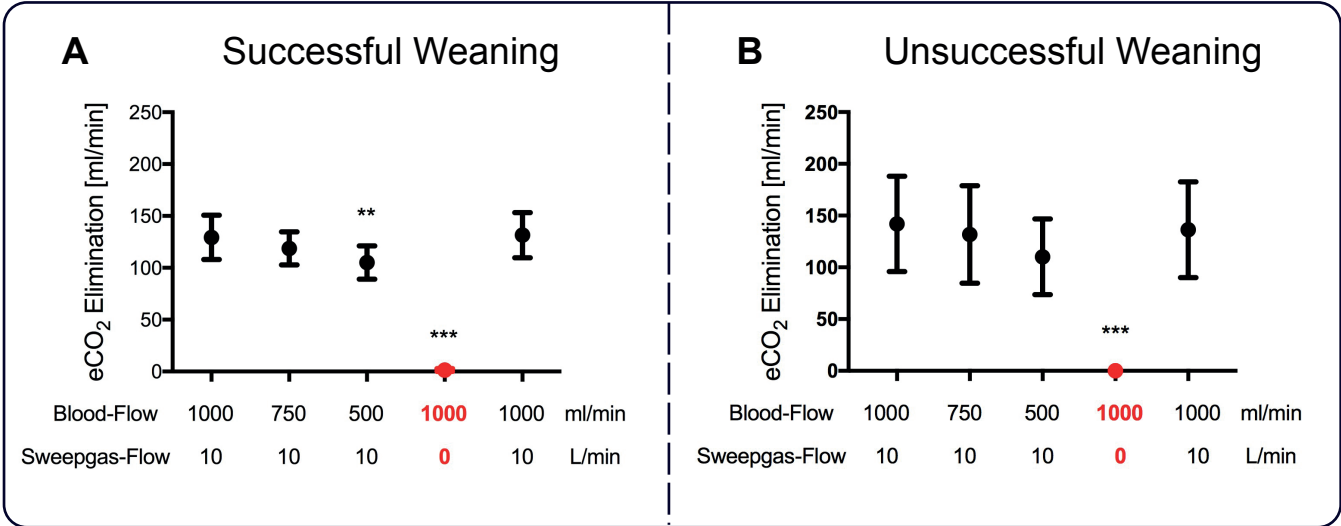

Supplement: Supplementary file 2 — Figure S1. Veno-venous extracorporeal CO2 elimination. First baseline extracorporeal CO2 removal was 129 ± 21 ml/min in the successful group (panel A) and 142 ± 46 mL/min in the unsuccessful group. From left to right, values obtained during first baseline (blood flow = 1000 mL/min and sweep gas flow = 10 L/min) and at blood flow to 750 mL/min and 500 mL/min with maintained sweep gas flow, followed by turning off sweep gas flow with 1000 mL/min blood flow and a second repeated baseline. For detailed description, see the main text. *Difference compared to baseline (*P < 0.05, **P < 0.01, ***P < 0.001). (PDF 1887 kb) [file 13054_2019_2404_MOESM2_ESM.pdf]

Supplemental Figure 3

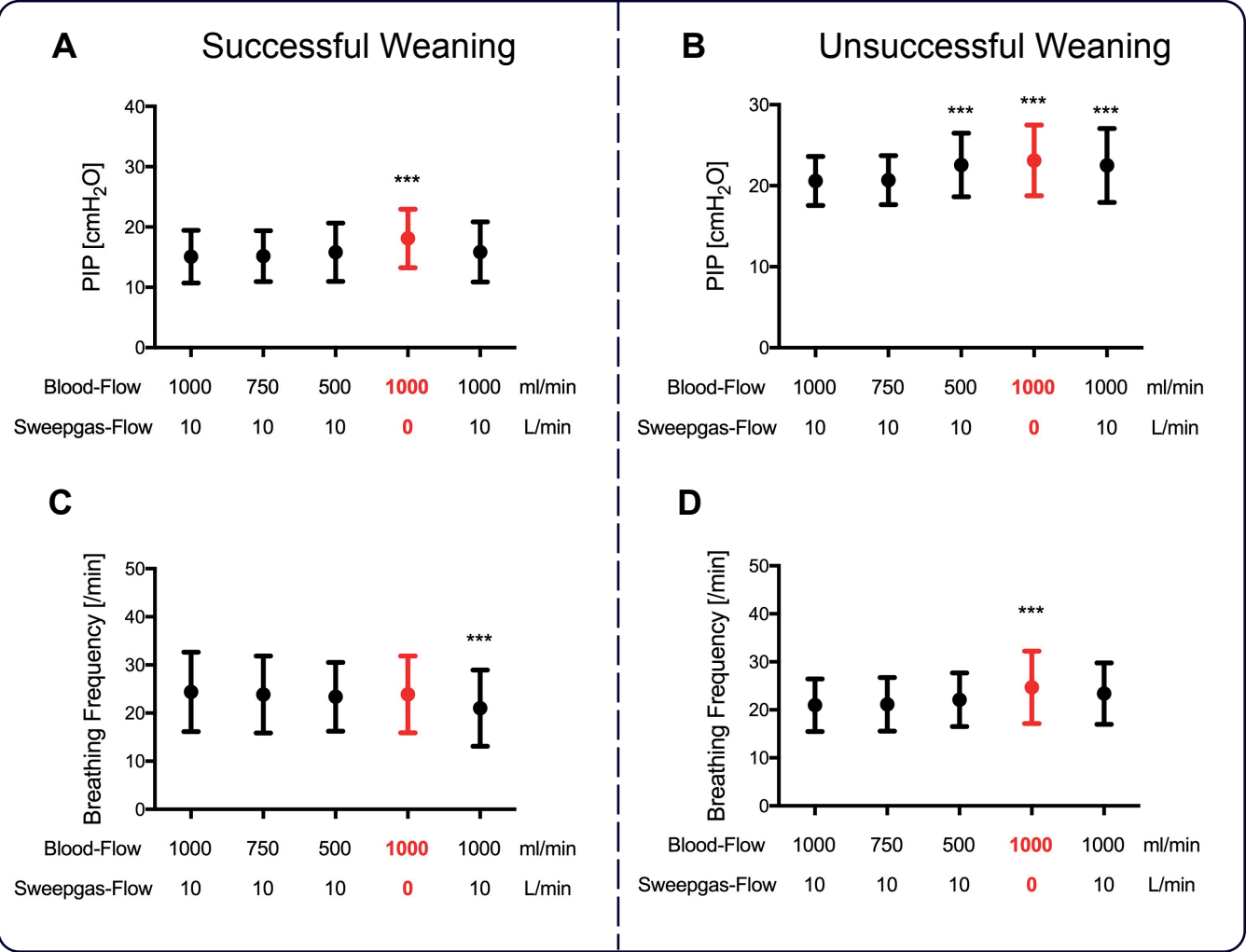

Supplement: Supplementary file 3 — Figure S3. Peak inspiratory airway pressure (PIP) and breathing frequency in (Fb) groups of successful and unsuccessful vv-ECCO2R weaning readiness test. From left to right, values obtained during first baseline (blood flow = 1000 ml/min and sweep gas flow = 10 L/min) and at blood flow to 750 ml/min and 500 mL/min with maintained sweep gas flow, followed by turning off sweep gas flow with 1000 mL/min blood flow and a second repeated baseline. For detailed description, see the main text. *Difference compared to baseline (*P < 0.05, **P < 0.01, ***P < 0.001). (PDF 2824 kb) [file 13054_2019_2404_MOESM3_ESM.pdf]

Supplemental Figure 2

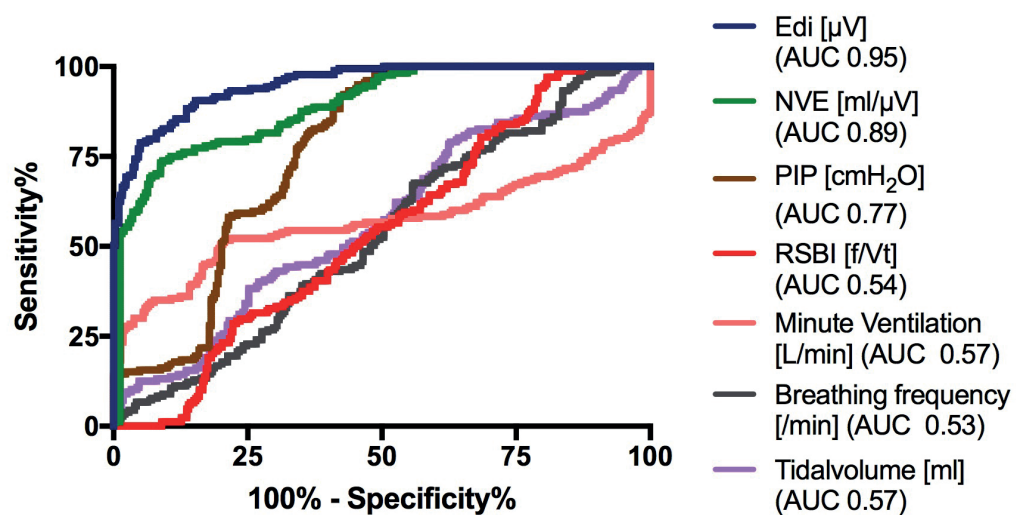

Supplement: Supplementary file 4 — Figure S2. Receiver-operating characteristic curve (ROC) analysis for the groups of successful and unsuccessful vv-ECCO2R weaning readiness test (N = 20) that were ventilated on NIV-NAVA when sweep gas flow was turned off. ROC analysis was applied for peak diaphragm electrical activity (Edi), neuro-ventilatory efficiency (NVE), peak airway pressure (PIP), rapid shallow breathing index (RSBI), minute ventilation, breathing frequency, and tidal volume. For detailed description see the main text. (PDF 2343 kb) [file 13054_2019_2404_MOESM4_ESM.pdf]
